# Supplementary material for: Dynamic transcriptomic profiles of zebrafish gills in response to zinc supplementation
Source: BMC Genomics. 2010 Oct 11;11:553. doi: 10.1186/1471-2164-11-553 (PMC3091702; doi:10.1186/1471-2164-11-553)
Supplement: Additional file 2 — Interactive Direct Interaction Network representing the molecular interactions between zinc, copper, iron, calcium and proteins encoded by transcripts changed by zinc supplementation. Mini web-site containing index.html and hyperlinked pages in subdirectory describing a Direct Interaction Network automatically generated based on curated interactions contained within the proprietary PathwayArchitect database. Ovals represent proteins and the circles symbolize metal ions. Objects are coloured by their abundance in zebrafish at the time-point they were significantly different from the control is a scale from -4 fold (dark green) to +4 fold (dark red). Where significant differences were found at more than one time-point, the colour overlay shows expression at the first instance. Dark blue squares denote 'binding', and light blue squares 'expression'; green squares stand for 'regulation', green diamonds for 'metabolism', and green circles for 'promoter binding'. Arrow heads indicate directionality of the interaction where annotated. All nodes and edges can be further interrogated by selecting the relative area of the image. [file 1471-2164-11-553-S2.zip › PathwayArchitect Zn xs DIN/1594415.html]

# BINDING:

|  |  |
| --- | --- |
| Type | BINDING |
| Effect | None |


---

|  |  |
| --- | --- |
| Score | 0 |


---

|  |  |
| --- | --- |
| Reference Count | 10 |


---

|  |  |
| --- | --- |
| Mechanism | Unknown |


---

|  |  |
| --- | --- |
| Reference:0 || PMID | 10074939 |
| SourceID | 229356 |
| Species | Human |
| Experimental Condition | in-vitro |
| Description | The three dimensional structure of Collagenase-3 (Mmp-13) Complexed to A Diphenyl-Ether Sulphone Based Hydroxamic Acid was determined by crystallography. The crystal was grown by vapour diffusion using Mmp-13, pH 8.0, at 100K. X-ray data were collected using radiation and were 90.6% complete to a resolution limit of 2.4 angstroms and with an overall Rsym of 0.0%. This crystal belonged to the space group C 1 2 1. The structure was determined using Molecular Replacement and the modeled structure was refined to an R-factor = 21.6% and had an R-free = 33.8%. PDB: 456C, MMDB: 11046. |
| Detection Method | three-dimensional-structure |
| Source | BIND |
  |
|  |  |


---

|  |  |
| --- | --- |
 Reference:1 || PMID | 10074939 |
| SourceID | 229356 |
| Species | Human |
| Experimental Condition | in-vitro |
| Description | The three dimensional structure of Collagenase-3 (Mmp-13) Complexed to A Sulphone-Based Hydroxamic Acid was determined by crystallography. The crystal was grown by using Mmp-13, 10-14% Polyethylene Glycol 4,000 Or 8,000 1.0-1.5m Ammonium Formate and 100 mM Tris pH 8.5 pH 8.0, at 100K. X-ray data were collected using synchrotron radiation and were 91.0% complete to a resolution limit of 1.6 angstroms with an overall Rsym of 8.7%. This crystal belonged to the space group C 1 2 1. The structure was determined using Molecular Replacement and the modeled structure was refined to an R-factor = 20.8% and had an R-free = 26.6%. PDB: 830C, MMDB: 11066. |
| Detection Method | three-dimensional-structure |
| Source | BIND |
  ||  |  |


---

|  |  |
| --- | --- |
 Reference:2 || PMID | 10926524 |
| SourceID | 229356 |
| Species | Human |
| Experimental Condition | in-vitro |
| Description | The three dimensional solution structure of the Catalytic Domain of Human Collagenase-3 (Mmp-13) Complexed to A Potent Non-Peptidic Sulfonamide Inhibitor was determined by using NMR spectroscopy. Collagenase 3 was dissolved in a solution containing 0.5 mM Collagenase-3 15N, 13C, 20 mM Tris D11, 20 mM CaCl2, 0.02% Nan3, 5 mM DTT, 100 mM NaCl, 0.1 mM Zncl, 100 mM NaCl pH 6.8 at 310K. The NMR experiments conducted were 3D Hnca, 3D 15N Edited NOESY, 2D 15N HSQC, 2D 13C HMQC, 3D CBCA(CO)NH, 3D Hbha(CO)NH, 3D Hncacb, 3D HNCO, 15N Filtered NOESY, 2D 13C Filtered NOESY using field strength(s) of 600 MHz, 500 MHz. The structure was determined by Simulated Annealing and Energy Minimization. 50 structures were calculated and 20 structures were submitted. PDB: 1EUB, MMDB: 16298. |
| Detection Method | three-dimensional-structure |
| Source | BIND |
  ||  |  |


---

|  |  |
| --- | --- |
 Reference:3 || PMID | 10959634 |
| SourceID | 229356 |
| Species | Human |
| Experimental Condition | in-vitro |
| Description | The three dimensional solution structure of the Catalytic Fragment of Human Collagenase-3 (Mmp-13) Complexed with A Hydroxamic Acid Inhibitor was determined by using NMR spectroscopy. Collagenase-3 was dissolved in a solution containing 1mM U-15N,13C-Labeled mMp-13 with Way-151693 in A 1:1 Ratio,10mM, 10 mM Deuterated Tris- pH 6.5 at 308K. The structure was determined by Distance Geometry Simulated Annealing. PDB: 1FLS, MMDB: 16744. |
| Detection Method | three-dimensional-structure |
| Source | BIND |
  ||  |  |


---

|  |  |
| --- | --- |
 Reference:4 || PMID | 10959634 |
| SourceID | 229356 |
| Species | Human |
| Experimental Condition | in-vitro |
| Description | The three dimensional solution structure of the Catalytic Fragment of Human Collagenase-3 (Mmp-13) Complexed with A Hydroxamic Acid Inhibitor was determined by using NMR spectroscopy. Collagenase-3 was dissolved in a solution containing 1 mM of U-15N,13C mMp-13, 10 mM Deuterated Tris- pH 6.5 at 308K. The structure was determined by Distance Geometry Simulated Annealing. 100 structures were calculated and 30 structures were submitted based on the structures with Acceptable Covalent Geometry,structures with Favorable Non-Bond Energy,structures with the Least Restraint Violations,structures with the Lowest Energy, Target Function. PDB: 1FM1, MMDB: 16745. |
| Detection Method | three-dimensional-structure |
| Source | BIND |
  ||  |  |


---

|  |  |
| --- | --- |
 Reference:5 || PMID | 15780611 |
| SourceID | 229356 |
| Experimental Condition | in-vitro |
| Description | The three dimensional structure of the Catalytic Domain of Mmp-13 Complexed with A Potent Pyrimidinetrione Inhibitor was determined by crystallography. The crystal was grown by hanging-drop and vapour diffusion using Collagenase 3, X-ray data were collected using radiation and were 94.4% complete to a resolution limit of 2.060 angstroms. This crystal contained 2 chains per asymmetric unit and belonged to the space group P 21 21 2. The structure was determined using Molecular Replacement and the modeled structure was refined to an R-factor = 21.5% and had an R-free = 29.5%. PDB: 1YOU, MMDB: 32529. |
| Detection Method | three-dimensional-structure |
| Source | BIND |
  ||  |  |


---

|  |  |
| --- | --- |
 Reference:6 || PMID | 10909870 |
| SourceID | 238907 |
| Experimental Condition | in-vitro |
| Description | The three dimensional solution structure of A Catalytic Domain of Mmp-2 Complexed with Sc-74020 was determined by using NMR spectroscopy. Matrix Metalloproteinase-2 was dissolved in a solution containing 0.3-0.4mM U-15N, 13C mMp-2c Unlabeled Sc-74020 in 20mM Tris-D11-HCl, 5mM CaCl2, 10 Zncl2, 20um Unlabeled Sc-74, 50 pH 7.4 at 303K. The NMR experiments were conducted using field strength(s) of 600 MHz, 500 MHz. The structure was determined by Distance Geometry, Simulat Annealing, Molecular Dynami Torsion Angle Dynamics. 14 structures were calculated and 14 structures were submitted based on the structures with the Least Restraint Violations. PDB: 1HOV, MMDB: 18195. |
| Detection Method | three-dimensional-structure |
| Source | BIND |
  ||  |  |


---

|  |  |
| --- | --- |
 Reference:7 || PMID | 10909870 |
| SourceID | 238908 |
| Experimental Condition | in-vitro |
| Description | The three dimensional solution structure of A Catalytic Domain of Mmp-2 Complexed with Sc-74020 was determined by using NMR spectroscopy. Matrix Metalloproteinase-2 was dissolved in a solution containing 0.3-0.4mM U-15N, 13C mMp-2c Unlabeled Sc-74020 in 20mM Tris-D11-HCl, 5mM CaCl2, 10 Zncl2, 20um Unlabeled Sc-74, 50 pH 7.4 at 303K. The NMR experiments were conducted using field strength(s) of 600 MHz, 500 MHz. The structure was determined by Distance Geometry, Simulat Annealing, Molecular Dynami Torsion Angle Dynamics. 14 structures were calculated and 14 structures were submitted based on the structures with the Least Restraint Violations. PDB: 1HOV, MMDB: 18195. |
| Detection Method | three-dimensional-structure |
| Source | BIND |
  ||  |  |


---

|  |  |
| --- | --- |
 Reference:8 || PMID | 9020046 |
| SourceID | 232548 |
| Species | Mouse |
| Experimental Condition | in-vitro |
| Description | The three dimensional structure of Recombinant Mouse Collagenase-3 (Mmp-13) was determined by crystallography. The crystal was grown by using Collagenase-3, X-ray data were collected using radiation and were 85.9% complete. This crystal contained 2 chains per asymmetric unit and belonged to the space group P 1 21 1. The structure was determined using the Single Wavelength diffraction protocol and the modeled structure was refined to an R-factor = 19.6% and had an R-free = 23.4%. PDB: 1CXV, MMDB: 14023. |
| Detection Method | three-dimensional-structure |
| Source | BIND |
  ||  |  |


---

|  |  |
| --- | --- |
 Reference:9 || PMID | 9020046 |
| SourceID | 232549 |
| Species | Mouse |
| Experimental Condition | in-vitro |
| Description | The three dimensional structure of Recombinant Mouse Collagenase-3 (Mmp-13) was determined by crystallography. The crystal was grown by using Collagenase-3, X-ray data were collected using radiation and were 85.9% complete. This crystal contained 2 chains per asymmetric unit and belonged to the space group P 1 21 1. The structure was determined using the Single Wavelength diffraction protocol and the modeled structure was refined to an R-factor = 19.6% and had an R-free = 23.4%. PDB: 1CXV, MMDB: 14023. |
| Detection Method | three-dimensional-structure |
| Source | BIND |
  |


---

|  |  |
| --- | --- |
